# Epidemiological Characteristics, Antibiotic Resistance Patterns and Genomic Analysis of *Shigella* Isolated in Pudong, Shanghai During 2013–2024

**DOI:** 10.3390/antibiotics14111091

**Published:** 2025-10-30

**Authors:** Yue Zhang, Xiao Wang, Yanru Liang, Wenqing Wang, Hong Huang, Bowen Yang, Anran Zhang, Yuan Zhuang, Min Chen, Jun Feng, Bing Zhao

**Affiliations:** 1 Shanghai Pudong New Area Center for Disease Control and Prevention, Shanghai Pudong New Area Health Supervision Institute, Shanghai 200136, China; yue7757199@gmail.com (Y.Z.); wangxiaoyouran@126.com (X.W.); lyr1985254806@163.com (Y.L.); wwq95@163.com (W.W.); 15026923993@163.com (H.H.); 18916161522@163.com (B.Y.); cdczhanganran@126.com (A.Z.); 2 National Institute of Pathogen Biology, Chinese Academy of Medical Sciences and Peking Union Medical College, Beijing 100730, China; 3 Shanghai Municipal Center for Disease Control and Prevention, Shanghai 201106, China; zhuangyuan@scdc.sh.cn (Y.Z.); chenmin@scdc.sh.cn (M.C.)

**Keywords:** *Shigella*, antimicrobial resistance, resistance gene, virulence factor, phylogeny

## Abstract

**Background:** *Shigella* spp. are critical pathogens causing diarrheal diseases. This study analyzed the epidemiological characteristics, antimicrobial resistance profiles, virulence factor profiles, and molecular patterns of *Shigella* isolates in Pudong New Area, Shanghai, from 2013 to 2024. **Materials and Methods:** Antimicrobial susceptibility of *Shigella* isolates was determined using the broth microdilution method. All molecular characterization analyses were based on whole-genome next-generation sequencing of *Shigella* strains. **Results:** A total of 55 *Shigella* spp. isolates were obtained from 17,670 enrolled diarrheal cases between 2013 and 2024, including 47 *S. sonnei* and 8 *S. flexneri* isolates. Resistance rates to sulfamethoxazole-trimethoprim (SXT), streptomycin (STR), nalidixic acid (NAL), ampicillin (AMP), and tetracycline (TET) all exceeded 90.00%. The resistance rate to azithromycin (AZI) increased from 12.50% to 60.00% with a fluctuating upward trend from 2013 to 2019; 97.87% of *S. sonnei* and 100.00% of *S. flexneri* isolates were multidrug-resistant. These isolates harbored multiple resistance genes and virulence factors. *S. sonnei* was dominated by ST152, while *S. flexneri* was predominantly ST245. These isolates were phylogenetically close to domestic (Beijing) and international (USA) strains of the same sequence typing collected at different time points, suggesting a common origin and stable transmission characteristics. **Conclusion:** From 2013 to 2024, the prevalent *Shigella* species in Pudong were *S. sonnei* and *S. flexneri*. *Shigella* isolates exhibited high resistance rates, and the situation of multidrug resistance was severe. Therefore, strengthening antimicrobial resistance monitoring and controlling regional transmission are of great significance. Meanwhile, genomic surveillance of *Shigella* ST152/ST245 is recommended for Pudong’s enteric pathogen control programs.

## 1. Introduction

*Shigella* spp. are well-recognized as significant enteric pathogens causing diarrheal diseases, which manifest as bacillary dysentery in humans, characterized by clinical symptoms such as diarrhea, fever, and tenesmus. Globally, an estimated 188 million cases of *Shigella* infection and 1.1 million related deaths are reported annually [1]. Taxonomically, *Shigella* is classified into four distinct serogroups (*Shigella sonnei*, *Shigella flexneri*, *Shigella dysenteriae*, and *Shigella boydii*) based on surface antigenic properties and biochemical characteristics [2]. *S. sonnei* predominates in developed countries, whereas *S. flexneri* is more endemic in developing nations. *S. boydii* and *S. dysenteriae* infections remain relatively rare. However, emerging evidence indicated that the prevalence of *S. sonnei* is gradually rising in developing countries, even replacing *S. flexneri* as the dominant species [3].

In China, *Shigella* constitutes a significant public health burden, with infection rates remaining notably higher than those in many developed areas. The epidemiology of *Shigella* infection exhibited pronounced spatiotemporal and demographic variations. For instance, distinct epidemiological profiles have been documented in Beijing, Shanxi Province, and Fujian Province [1,4,5,6]. Pudong New Area of Shanghai is located in a warm and humid coastal area, characterized by dense population and rich domestic and international transportation networks, facing the potential risk of *Shigella* transmission at all times.

Antibiotics are commonly used in clinical practice to treat bacillary dysentery. However, the irrational use of these drugs has led to a continuous increase in the drug resistance of *Shigella* [7,8]. Previous studies have shown that *Shigella* displayed widespread resistance to commonly used antibiotics such as ampicillin and tetracycline globally. In addition, the emergence of resistance to quinolones, cephalosporins, and sulfonamides should not be overlooked [9]. In 2024, the World Health Organization (WHO) updated its list of priority bacterial pathogens, elevating fluoroquinolone-resistant *Shigella* from “medium” to “high” priority, which implies the severe situation of *Shigella* antibiotic resistance [10]. Without exception, drug-resistant *Shigella* strains, even multidrug-resistant isolates, have been reported in different regions of China. The resistance mechanisms of these *Shigella* strains are mainly involved in the presence or mutation of various resistance genes. Timely clarification of the resistance patterns and potential mechanisms of *Shigella* contributes to guiding disease treatment and preventing its spread within the population.

As China’s economic hub with extensive international connectivity, Pudong’s dense population and high mobility may accelerate the spread of antimicrobial-resistant *Shigella*, yet longitudinal data remains scarce. Therefore, this study explored the epidemiological characteristics and antibiotic resistance profiles of *Shigella* in this region during 2013–2024. Whole-genome sequencing technology was employed to comprehensively characterize the carriage of virulence factors and antibiotic resistance genes. Meanwhile, phylogenetic tree construction was performed to decipher the molecular evolutionary characteristics of *Shigella* throughout the study period, with the aim of guiding public health prevention and control strategies against *Shigella* infections.

## 2. Results

### 2.1. Identification of Isolates and Prevalence of Shigella spp.

**Figure 1:**
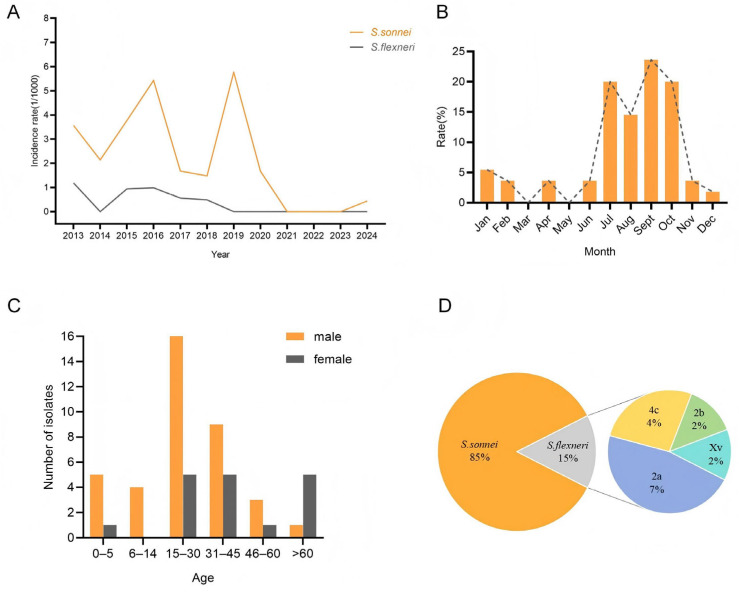
Basic epidemiological characteristics of *Shigella* in Pudong, Shanghai from 2013 to 2024. (**A**) The line chart shows the incidence rate (1/1000) of *S. sonnei* (marked in yellow) or *S. flexneri* (marked in gray); (**B**) Monthly distribution of *Shigella* infections; (**C**) Age and sex distribution of *Shigella* cases; (**D**) Serotypes distribution of *Shigella* isolates.

During the study period, 55 (3.11‰) *Shigella* spp. Isolates were obtained from 17,670 enrolled diarrheal cases. The total number of biological samples collected during 2013–2019 remained relatively stable, while the positive rate of *Shigella* showed wave-like fluctuations, peaking at 6.42‰ in 2016 and reaching the lowest of 1.98‰ in 2018 (Table 1). However, affected by the COVID-19 pandemic, the number of collected samples began to decrease since 2020, accompanied by a significant decline in the positive rate of *Shigella*, which even reached 0 (Figure 1A).

Figure 1B displayed the monthly isolation profile of all *Shigella* strains. The detection rate of *Shigella* exhibited distinct seasonal characteristics, with the highest rates observed in summer and autumn (July–October) and consistently low levels during winter and spring. Figure 1C illustrated that *Shigella*-infected populations also showed remarkable gender and age-specific patterns. The majority of infected individuals were distributed in the 15–45 age group, accounting for 63.64% (35/55) of the total infected population. Males outnumbered females in almost every age group, except for the >60 years old group.

Typing analysis of the obtained *Shigella* strains showed that all isolates belonged to two serogroups: *S. sonnei* and *S. flexneri*. Among them, 47 strains (85.45%) were identified as *S. sonnei*, and 8 strains (14.55%) as *S. flexneri*. Neither *S. dysenteriae* nor *S. boydii* were detected. Overall, *S. sonnei* was the dominant species in Pudong District. Further serotyping revealed that *S. flexneri* isolates comprised four serotypes: 2a, 4c, 2b, and Xv, with serotype 2a being the most prevalent (Figure 1D).

### 2.2. Antimicrobial Resistance in Shigella spp.

**Figure 2:**
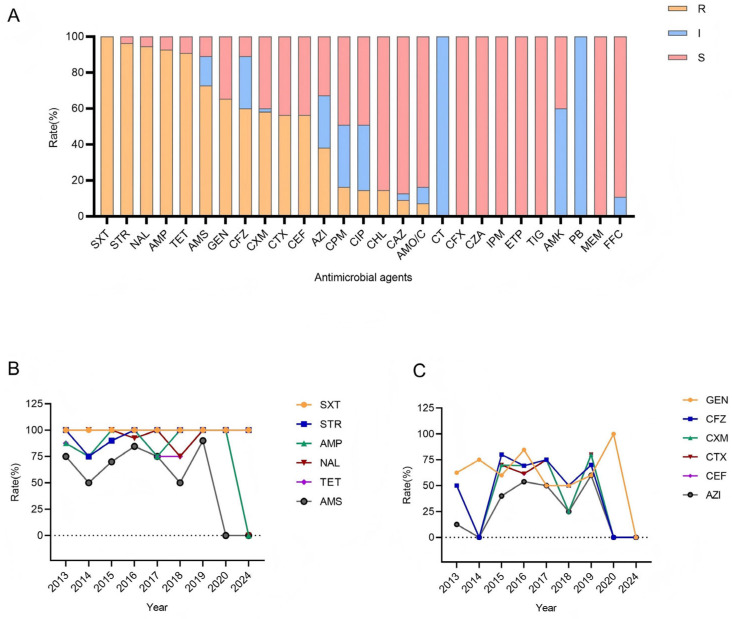
Antimicrobial resistance rates of *Shigella* isolates to different antimicrobial agents in Pudong, Shanghai from 2013 to 2024. (**A**) Proportions of all *Shigella* isolates that were susceptible (S, marked in red), intermediate (I, marked in blue), or resistant (R, marked in yellow) to antimicrobial agents. (**B**) Trends in antimicrobial resistance rates of *Shigella* isolates to SXT, STR, AMP, NAL, TET and AMS from 2013 to 2024. (**C**) Trends in antimicrobial resistance rates of *Shigella* isolates to GEN, CFZ, CXM, CTX, CEF and AZI from 2013 to 2024 (CTX and CEF lines overlap owing to high data similarity).

The antimicrobial resistance profiles of all *Shigella* isolates against 27 antibiotics were presented in Table 2. Data showed that the 55 *Shigella* isolates exhibited varying degrees of resistance to different antibiotics (Figure 2A). Resistance rates to trimethoprim/sulfamethoxazole (SXT), streptomycin (STR), nalidixic acid (NAL), ampicillin (AMP), and tetracycline (TET) all surpassed 90.00%, with the sequence of their resistance rates being 100.00%, 96.36%, 94.55%, 92.73%, and 90.91%. Resistance rates to ampicillin/Sulbactam (AMS), gentamicin (GEN), cefazolin (CFZ), cefuroxime (CXM), cefotaxime (CTX), and cefotiofur (CEF) were 72.73%, 65.45%, 60.00%, 58.18%, 56.36%, and 56.36%, respectively, all of which were over 50.00%. Serotype-specific analysis revealed significant differences in resistance patterns between *S. sonnei* and *S. flexneri*. *S. flexneri* showed significantly higher resistance rates to ciprofloxacin (CIP), chloramphenicol (CHL), and amoxicillin-clavulanic acid (AMO/C) than *S. sonnei* (*p* < 0.05). *S. sonnei* exhibited higher resistance to CTX and CEF than *S. flexneri*, but this difference was not statistically significant, possibly due to the limited sample size (Table 2). Temporally, from 2013 to 2019, resistance rates of to SXT, STR, NAL, AMP, and TET remained consistently high (75.00–100.00%), while isolates remained sensitive to colistin (CT), cefoxitin (CFX), ceftazidime/avibactam (CZA), imipenem (IPM), ertapenem (ETP), tigecycline (TIG), amikacin (AMK), polymyxin B (PB), meropenem (MEM), and florfenicol (FFC). Crucially, the resistance rate of *Shigella* isolates to azithromycin (AZI) increased from 12.50% to 60.00%, showing a fluctuating upward trend (Figure 2B,C).

Multiple Drug Resistance (MDR) is defined as resistance to three or more classes of antibiotics. In this study, 98.18% (*n* = 54) of isolates exhibited MDR. Specifically, 97.87% (*n* = 46) of *S. sonnei* and 100% (*n* = 8) of *S. flexneri* isolates were MDR. The most prevalent resistance patterns among all isolates were AMP/AMS/CFZ/CTX/CXM/SXT/NAL/GEN/TET/STR/CEF (*n* = 13), AMP/AMS/SXT/NAL/GEN/TET/STR (*n* = 7), and AMP/SXT/NAL/GEN/TET/STR (*n* = 4).

To elucidate the mechanisms of antimicrobial resistance, the carriage of resistance genes in *Shigella* isolates was investigated (Table 3). The prevalence of antibiotic resistance-related genes varied significantly. Overall, the most frequently detected resistance genes were *dfrA1* (98.18%), *gyrA* (S83L) (96.36%), *ant(3″)-Ia* (80.00%), and *tet(A)* (69.09%), which are associated with resistance to macrolides, trimethoprim, fluoroquinolones, aminoglycosides, and tetracyclines, respectively. The carriage rates of *tet(A)*, *ant(3″)-Ia*, and *sul2* were significantly higher in *S. sonnei* than in *S. flexneri*, whereas *S. flexneri* exhibited significantly higher prevalence of *tet(B)* and *parC* (S80I) compared to *S. sonnei*.

According to the literature and clinical practice, nine antibiotics were chosen to analyze the consistency between drug resistance phenotypes and genotypes. The findings indicated that a total of 495 phenotypes were obtained from 55 *Shigella* isolates, among which 360 phenotypes were consistent with genotypes, with an overall consistency rate of 72.73% (Table 4). Among 357 isolates exhibiting resistant phenotypes, 130 cases (36.41%) lacked relevant resistance genes, which was predominantly observed in AMP (penicillin drug), CTX (cephalosporin drug) and AZI (macrolide drug). Conversely, among 138 isolates with susceptible/intermediate phenotypes, 5 cases (3.62%) harbored related resistance genes. Specifically, the resistance genes for TET, STR, SXT and NAL demonstrated high sensitivity (>80%). Those related to GEN and CIP exhibited moderate sensitivity (66.67% and 62.5%, respectively), while the sensitivity of AMP, CTX and AZI associated resistance genes was poor (<10%).

### 2.3. Virulence Factors

**Figure 3:**
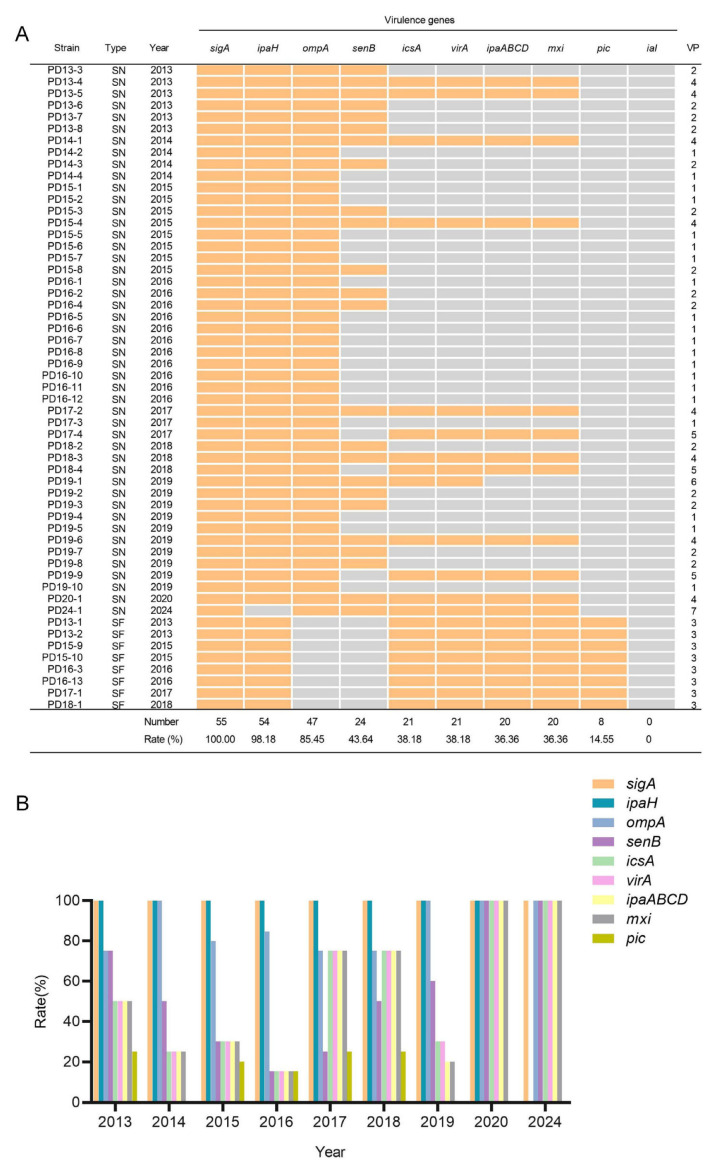
Distribution of virulence factors in *Shigella* isolates. (**A**) Presence (marked in yellow) or absence (marked in gray) of virulence factors in *Shigella* isolates. SN, *S. sonnei*; SF, *S. flexneri*; VP, virulence factor profiles. (**B**) Trends in the virulence factors carried by *Shigella* from 2013 to 2024.

A total of 10 key virulence factors of *Shigella* (*sigA*, *ompA*, *ipaH*, *senB*, *icsA*, *virA*, *ipaABCD*, *mxi*, *pic*, *ial*) were focused on (Figure 3). All isolates harbored factors *sigA*, but none carried *ial*. The carriage rates of other virulence factors in 55 *Shigella* strains were 98.18% (*ipaH*), 85.45% (*ompA*), 43.64% (*senB*), 38.18% (*icsA*), 38.18% (*virA*), 36.36% (*ipaABCD*), 36.36% (*mxi*) and 14.55% (*pic*). A single strain of *S. sonnei* lacked the characteristic *ipaH* gene. Among them, *ompA* and *senB* were only present in *S. sonnei*, while the *pic* factor was only present in *S. flexneri*. For *S. flexneri*, the types of virulence factors carried by strains isolated from 2013 to 2024 remained unchanged. In the case of *S. sonnei*, the presence rate of the enterotoxin-encoding *senB* gene gradually decreased from 2013 to 2017 (from 100% to 18.19%) and then showed an upward trend after 2018. The detection status of virulence factors *icsA*, *virA*, *ipaABCD*, and *mxi* remained consistent. The overall presence rate of these four factors was relatively low from 2013 to 2016 and showed a year-by-year decreasing trend, but a sudden increase was observed starting from 2017. These strains collectively formed 7 virulence factor profiles (VP), which were listed in Table 5.

### 2.4. Phylogenetic Analysis

Multilocus sequence types (MLST) analysis was conducted to characterize the evolutionary dynamics of *Shigella* isolates throughout the study period. Simultaneously, SNP-based phylogenetic trees were constructed using the 55 study isolates (47 *S. sonnei* and 8 *S. flexneri*) in conjunction with reference sequences retrieved from public databases (28 *S. sonnei* and 20 *S. flexneri* strains).

The MLST analysis revealed three distinct sequence types (STs) among 55 *Shigella* strains. Within *S. sonnei*, two STs were recognized: ST152 was the predominant type, comprising 43 strains (91.49%), while the remaining 4 strains (8.51%) belonged to ST6919. Importantly, all *S. sonnei* isolates collected from 2013 to 2018 were exclusively ST152, with ST6919 emerging as a different type in 2019. In contrast, all *S. flexneri* strains collected across all sampling years were uniformly assigned to ST245.

**Figure 4:**
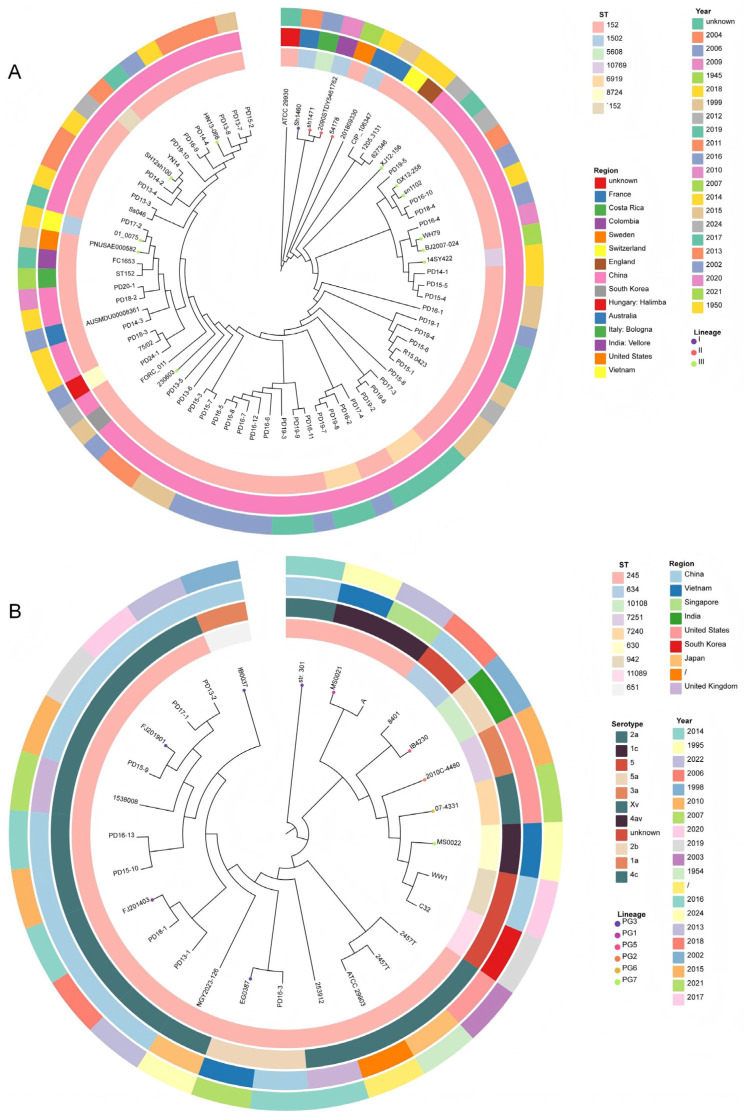
Phylogenetic tree for core-genome SNP of *S. sonnei* isolates (**A**) and *S. flexneri* isolates (**B**) in a global context. The annotations (**A**) were (from inner to outer ring): ST, region and year of *S. sonnei* isolates. Lineages I–III were marked as circular dots of different colors on the names of leaves. The annotations (**B**) were (from inner to outer ring): ST, serotype, region and year of *S. flexneri* isolates. Phylogenetic groups (PG) 1–7 were marked as circular dots of different colors on the names of leaves.

All *S. sonnei* isolates were categorized into Lineages I–III. All local isolates and a substantial proportion of other Chinese strains clustered within Lineage III, forming a distinct Chinese evolutionary clade. Within this lineage, ST152 was the dominant sequence type among the 47 local isolates, but it was also detected in strains collected from South Korea, Australia, Italy, India, and the United States over different time periods, suggesting the potential for intercontinental spread of the ST152 clone. Phylogenetic analysis unveiled that *S. sonnei* strains from Shanghai were genetically close to international strains and domestic isolates from Wuhan (WH79), Beijing (BJ2007-024), and Yunnan (YN14), indicating a common ancestry (Figure 4A).

All *S. flexneri* isolates belonged to multiple phylogenetic groups (PG1-PG7, excluding PG4). The majority of Chinese strains, including the 8 local isolates, clustered within PG3. With the exception of IB0037, all PG3 strains were assigned to ST245. Serotyping analysis revealed diverse serotypes within PG3, including 2a, 4C, 1a, and Xv, with 2a having the highest frequency, suggesting a potential association between PG3 and serotype 2a. Geographically and temporally, PG3 encompassed strains from Vietnam, Japan, and the United Kingdom, spanning approximately two decades, indicating long-term colonization and stable transmission across regions. Shanghai isolates grouped within the same major clade as international strains and neighboring Fujian isolates (FJ201403 and FJ201901), suggesting multiple origins and strong adaptability of local strains (Figure 4B).

## 3. Discussion

*Shigella* is an infectious intestinal pathogen and ranks among the primary etiologies of diarrheal diseases in developing countries. It can cause acute enteritis, presenting patients with symptoms such as watery diarrhea, high fever, and bloody stools, thus posing a critical public health concern [11,12]. Based on diarrheal disease surveillance samples collected from Pudong New Area between 2013 and 2024, this study systematically explored the infection rate, temporal distribution, serotyping, as well as the type and age characteristics of infected populations of *Shigella* in the region. It further clarified the drug-resistant phenotypes, resistance mechanisms, virulence gene carriage, and molecular evolution characteristics of *Shigella*, thereby providing data support for the prevention and control of *Shigella*.

The positive rate of *Shigella* in Pudong New Area ranged from 1.98‰ to 6.428‰ between 2013 and 2019, remaining at a relatively low overall level. After 2019, almost no *Shigella* was detected, which may be attributed to home quarantine measures, enhanced personal hygiene, and significantly improved protection awareness implemented during the COVID-19 pandemic. A study investigating pathogens in patients with acute diarrhea in China from 2009 to 2018 identified *Shigella* as an important bacterial pathogen with a detection rate of 6.88% [13], whereas the detection rate in this region was much lower. This discrepancy might be related to factors such as local political and economic development, protection of water source environments, and the level of public health services. *Shigella* epidemics exhibited distinct seasonal and population-specific characteristics. In this region, *Shigella* prevalence was mainly concentrated in July–October, which is similar to the overall epidemic period in China (June–September) [14]. The prevalence in Pudong New Area during this period was also associated with the high temperature and humidity typical of this southern coastal area. Such climatic conditions could affect bacterial reproduction rates, leading to seasonal fluctuations in infection rates [15,16]. Consequently, future surveillance efforts should focus on this time window to prevent *Shigella* disease outbreaks. The results of this study showed that more male than female individuals were infected with *Shigella*, covering all age groups with a concentration in the 15–45 years range. However, numerous studies have indicated that *Shigella* tends to affect children and the elderly more frequently [12,17]. The following reasons may account for this discrepancy. First, children with *Shigella* infection may present with mild symptoms and thus do not seek medical attention, resulting in underreporting. Second, the young and middle-aged population, spanning 15 to 45 years of age, engages in social activities and interpersonal interactions with greater frequency and over a broader range compared to children or the elderly, which directly heightens their susceptibility to *Shigella* infection. *Shigella flexneri* and *Shigella sonnei* were the two most prevalent serotypes in developing countries [18]. Typically, *Shigella flexneri* was the predominant isolate in developing regions, such as Peru in Latin America, as well as Bangladesh, India and other South Asian countries [19,20,21,22,23]. In contrast, *Shigella sonnei* was more common in developed countries. Consistent with the findings of this study, *Shigella sonnei* was the dominant serotype in domestic cities including Beijing, Fujian, and Guizhou [1,5,24]. In some newly industrialized regions such as Thailand, South Korea and Taiwan, *Shigella sonnei* had also gradually replaced *Shigella flexneri* as the predominant strain [25]. These observations suggested that in addition to climate factors, the prevalence of *Shigella* was closely associated with the level of socioeconomic development. As a result, vaccine development against *Shigella* should focus on the main local *Shigella* serotypes to maximize the effectiveness of such vaccines.

Antibiotics are effective in treating *Shigella* infections, as they can significantly shorten the course of the disease [26]. Nevertheless, numerous studies have indicated that *Shigella* was progressively developing drug resistance, with even the emergence of multidrug-resistant strains, which posed a significant threat to the treatment of shigellosis. The patterns of drug resistance in *Shigella* vary by region and serotype; clarifying the resistance patterns in a specific area is conducive to guiding the formulation of shigellosis treatment regimens and prevention/control policies. Results of this study revealed that all 55 *Shigella* strains isolated in Pudong New Area from 2013 to 2024 were resistant to antibiotics, with no extreme fluctuations in the resistance rates to most antibiotics. Currently, the most commonly used drugs for *Shigella* infections were fluoroquinolones, AZI, and third-generation cephalosporins [1]. The quinolone NAL was once widely used for *Shigella* due to its efficacy and affordability, but as resistance has increased, it has gradually fallen out of favor as a recommended treatment. This study demonstrated that the resistance rate of *Shigella* to NAL in Pudong New Area was as high as 94.55%, higher than that in Kolkata, India (91.8%) and Jiangsu Province, China (90.5%) [23,27], but lower than that in Taiyuan, Shanxi Province (100%). These indicate a high overall resistance level to NAL, and its clinical use should be cautious. CIP was an improved key fluoroquinolone. A significant difference (*p* < 0.05) was observed in the resistance rates to CIP between *S. sonnei* and *S. flexneri*, at 4.26% and 75%, respectively. The overall resistance rate was 14.55%, lower than that in Kolkata, India (83.4%), Taiyuan, Shanxi Province (73.39%), Ningbo, Zhejiang Province (36%) and Fujian Province (21.67%) [28]. Thus, this may be a relatively favorable figure for this region, and CIP could be considered as a therapeutic option in clinical practice based on serotyping. In addition, AZI and CTX were also commonly recommended for shigellosis treatment. Worryingly, the resistance rate to AZI in Pudong New Area fluctuated and increased from 12.50% to 60% between 2013 and 2019. AZI remained a viable option, but there was an urgent need to identify the causes of its resistance and rapidly screen for resistance, thereby better serving clinical practice. The resistance rate to CTX also reached 80% in 2019, exceeding that in neighboring Jiangsu Province and Fujian Province [1,29]. This may exert a significant adverse impact on the use of these two drugs, and this phenomenon warrants close attention. Furthermore, the strains in this study showed extremely high resistance rates (over 90%) to SXT, STR, AMP, and TET, with almost no downward trend over the 12-year study period. Near-ubiquitous resistance to thesis antibotics invalidated their empirical use in Pudong region. In particular, SXT, an antibiotic generally prone to resistance, showed an alarming 100% resistance rate, consistent with findings in Iran and exceeding that in the United States (83%) and Bangladesh [30,31,32].

To gain an in-depth understanding of the resistance mechanisms of *Shigella* strains, it was necessary to investigate the carriage of resistance genes and compare their consistency with the drug-resistant phenotypes of *Shigella*. *Shigella* resistance to fluoroquinolones was primarily caused by point mutations in genes encoding DNA gyrase and topoisomerase IV, such as *gyrA*, *gyrB*, and *parC* within the quinolone resistance-determining regions (QRDRs) [33,34]. Among the 52 NAL-resistant strains, the *gyrA* (S83L) mutation was detected in all, with a sensitivity of 100%. Among the 8 CIP-resistant strains, 5 exhibited both *gyrA* (S83L) and *parC* (S80I) mutations, resulting in a sensitivity of 62.5% and a specificity of 97.87%. The primary resistance genes associated with AZI were *mphA* and *ermB* [35]. It is noteworthy that none of the 21 AZI-resistant *Shigella* isolates in this study carried these resistance genes. Nevertheless, these isolates harbored multidrug efflux pump genes such as mdtE and mdtF, suggesting that the resistance mechanism may be related to the overexpression of these efflux pump genes. *blaTEM-1B*, *blaOXA-1*, and blaCTX are different types of β-lactamase genes, often located on plasmids, which are easily transmitted among intestinal pathogens through conjugative transfer. They confer resistance by synthesizing corresponding enzymes that hydrolyze various antibiotics such as AMP and CTX. Notably, the sensitivity of resistance genes to β-lactams (AMP and CTX) in this study was unsatisfactory. A review of the literature suggests possible reasons for this: first, the deletion or downregulated expression of *Shigella* outer membrane proteins (e.g., OmpF, OmpC) could reduce transmembrane penetration of antibiotics [36]; second, the activation of acrAB-tolC efflux pumps in *Shigella* can non-specifically expel multiple antibiotics, thereby reducing intracellular drug concentrations [37,38,39]. Naturally, the specific mechanisms require further experimental verification.

The pathogenicity of *Shigella* is closely associated with several key virulence factors. Studies have shown that both *ipaH* and *ial* were important molecules mediating epithelial cell penetration and intercellular spread [18]. Among the 55 strains in this study, none possessed the *ial* gene, whereas 54 strains harbored the *ipaH* gene. Reports have demonstrated that *ipaH* was clinically correlated with symptoms such as fever, abdominal pain, and vomiting [18]. *Shigella* enterotoxin 1 (ShET-1) and *Shigella* enterotoxin 2 (ShET-2) were important enterotoxins that could cause intestinal mucosal damage and watery diarrhea. ShET-1 was encoded by the chromosomal gene *set1A*/*set1B* and was primarily present in *S. flexneri*, but it was not detected in the strains from this study. ShET-2 was mainly encoded by *senB* [18]. Since 2018, the prevalence of *senB* in *S. sonnei* isolates from this area has been increasing, suggesting a potential rise in virulence and necessitating attention to symptomatic treatment. Additionally, *icsA*, *virA*, *ipaABCD*, and *mxi* were also facilitators of cell-to-cell spread, invasion, and intestinal colonization in *Shigella* [40,41,42,43]. The detection rates of these four factors showed an upward trend during 2017–2018, slightly declined in 2019, and resurged in the strains isolated in 2020 and 2024, which warrants further concern. Regarding the *pic* factor, it was absent in all *S. sonnei* isolates in this study but present in all *S. flexneri* strains, consistent with findings from Anhui Province. The presence of the *pic* gene has also been shown to significantly contribute to increased resistance to CIP and CHL [43]. Consequently, special precautions and optimized treatment regimens were imperative for infections caused by such *Shigella* strains.

Phylogenetic analysis has clarified the phylogenetic position of local *Shigella* isolates within the national and global context. By examining SNPs and analyzing the topology of the phylogenetic tree, global *S. sonnei* can be divided into 4 lineages (Lineage I–IV). Lineages I–III were distributed across multiple regions including Europe, Asia, Africa, and the Americas. Lineage IV consists of a single isolate from France, which has a genetic profile markedly different from the other lineages [44,45,46,47]. Among them, Lineage III was the most widely spread branch globally, and most Chinese strains belonged to this lineage. As expected, all *S. sonnei* strains from Pudong New Area, Shanghai in this study were assigned to Lineage III, which was consistent with the findings of Huang et al. on isolates from Fujian [1]. ST152 was the core clonal type of these isolates, which had been colonizing and transmitting locally for a long time and may have become the dominant strain due to adaptation to the local population’s immunity and environment. These local isolates shared the ST152 type with strains collected at different times from regions such as South Korea, the United States, Beijing, and Wuhan, indicating that the ST152 clonal group was not unique to Shanghai. ST152’s clustering with South Korea (1999) and United States strains (2015) implicates that the increased movement of people and goods, driven by the booming economic and trade activities as well as the prosperity of tourism, has led to cross-regional transmission mediated by trade or travel. The phylogenetic tree branch map further confirmed that local strains may share a common ancestor with these domestic and international Lineage III strains. Accordingly, as one of China’s most important import and export transportation hubs, Pudong New Area Airport should enhance the screening of diarrheal passengers arriving from endemic regions to prevent the introduction and widespread transmission of *Shigella*.

A study conducted by Connor et al. divided *S. flexneri* into 7 independent phylogenetic groups (PG1–PG7). Each PG was generally restricted to epidemic spread in specific regions [48,49,50]. All isolates from Pudong New Area in this study belonged to PG3, with ST245 as the dominant type. From the phylogenetic tree, it could be observed that ST245 had been colonizing for a long time and transmitting across time and regions, so its drug resistance and virulence evolution need to be monitored with emphasis. Local isolates of different serotypes were clustered in PG3, among which serotype 2a was dominant and forms a tight cluster, reflecting that local epidemic strains have differentiated within PG3 without large genetic branches caused by serotype switching. It is worth noting that similar to *S. sonnei*, the *S. flexneri* isolates in this study were closely related to strains from domestic and foreign regions such as Vietnam and Fujian, and they may be homologous clonal strains.

This study undoubtedly has its limitations. On the one hand, the total number of strains included in this study is insufficient, which may affect the analysis of the genomic diversity of *Shigella* in this region and lead to statistical biases. On the other hand, due to the long passage of time, epidemiological and clinical information of these *Shigella*-infected individuals, such as their occupation, activity area, exposure history, clinical symptoms, and medication history, was incomplete, thereby hindering in-depth analysis of the transmission routes, virulence, and drug resistance of *Shigella*.

## 4. Materials and Methods

### 4.1. Specimen Collection and Shigella Isolation

According to the surveillance protocol for diarrheal diseases in Pudong New Area, samples from 2013 to 2024 in the study were collected from cases related to diarrhea diagnosis in several sentinel hospitals in the jurisdiction. Stool samples were streaked onto xylosine lysine deoxycholate (XLD) and then incubated at 37 °C for 18–24 h. Select suspicious positive colonies and conduct double tube sugar biochemical experiments. Further perform API 20E strips (BioMérieux, Shanghai, China) experiments on colonies that meet biochemical characteristics to confirm positive strains. At the same time, a slide agglutination test was carried out using Diagnostic Sera for *Shigella* Kit (Ningbo Tianrun Biopharmaceutical Co., Ltd., Ningbo, China) to identify the serotype of *Shigella* isolates. All isolates were immersed in nutrient agar containing glycerol and stored at −80 °C in a refrigerator.

### 4.2. Antimicrobial Susceptibility Testing

AST Panel For Aerobic Gram-Negative bacilli (Fosun Diagnostics Technology Co., Shanghai, China) was used to determine the resistance profile of *Shigella* spp. Antimicrobial susceptibility testing detected minimum inhibitory concentrations (MICs) values of 27 types of antibiotics, including AMP, AMO/C, AMS, CFZ, CTX, CFX, cefepime (CPM), CXM, CZA, ceftazidime (CAZ), CEF, IPM, ETP, MEM, CT, PB, TET, TIG, GEN, AMK, STR, SXT, AZI, CHL, FFC, CIP and NAL. The *E. coli* ATCC^®^25922TM strain was utilized as the control strain for antimicrobial susceptibility testing. On each antimicrobial susceptibility testing plate, a series of antibiotic reagents with two-fold diluted concentrations were prepared for each antibiotic. By adding suspension of the bacteria mixed with nutrient broth, after incubation for 16–20 h, antimicrobial susceptibility test strips were read. MICs values were determined through data analysis, and the results were categorized as susceptible (S), intermediate (I), or resistant (R) based on the Clinical and Laboratory Standards Institute (CLSI) standards.

### 4.3. Whole Genome Sequencing (WGS)

*Shigella* strains were recovered from −80 °C and cultivated on blood agar plates. The pure cultures were sent to the third party service provider for sequencing of the framework diagram. Genomic nucleic acid was extracted using BG-Nege-96 fully automatic nucleic acid extraction instrument and the supporting reagents (Shanghai Bojie Medical Technology Co., Ltd., Shanghai, China).The sequence was performed on the DNA library generated by Whole-genome DNA Library Construction Kit (Shanghai Bojie Medical Technology Co., Ltd., Shanghai, China) using MGISEQ-T7 platform (MGI Tech Co., Ltd., Shanghai, China). All raw sequencing datasets demonstrated a Q30 score exceeding 93%, meeting stringent quality thresholds for downstream analyses. Fastp software (version 0.23.2) was applied to filter the offline data of each sample, removing adapter and low-quality reads. SPAdes software (version 3.15.5) was employed for reference-free assembly of bacterial samples. Kraken (version 2.1.2) was utilized to annotate the assembled data and non-target bacterial sequences were filtered out.

### 4.4. Bioinformatics Analysis

The presence or mutations of resistance genes in *Shigella* isolates were identified via ABRicate v1.0.1 program (https://github.com/tseemann/abricate, accessed on 5 June 2025) based on the CARD and ResFinder database, and virulence factors were analyzed based on the VFDB database. MLST were obtained from online website Center for Genomic Epidemiology (CGE, https://www.genomicepidemiology.org/services/, accessed on 15 July 2025), with allele assignments based on sequence comparison against the reference MLST schemes. The complete genomes of *Shigella* strains used for constructing phylogenetic trees were downloaded from the NCBI database. The reference strains for *S. sonnei* and *S. flexneri* were ATCC 29930 (NZ_CP026802) and str. 301 (NC_004337), respectively. Single nucleotide polymorphism (SNP) analysis of the target strains was performed using the Parsnp tool (v2.1.4). The maximum likelihood method was employed to construct the phylogenetic tree, which was then visualized and annotated using the Chiplot platform (https://www.chiplot.online/tvbot.html, accessed on 21 July 2025).

### 4.5. Statistical Analysis

SPSS statistical software (version 20) was performed for statistical analysis. The pearson chi-square test or fisher’s exact test was used for analyzing categorical variables. Two-sided and *p* < 0.05 was considered to be statistically significant.

## 5. Conclusions

This study systematically analyzed the epidemiological and molecular characteristics of *Shigella* in Pudong New Area, Shanghai, from 2013 to 2024. The overall *Shigella* infection rate in this region was relatively low, with a peak in 2016 at 6.42‰. However, the antibiotic resistance of these *Shigella* isolates cannot be ignored, as they exhibited high resistance rates to commonly used antibiotics such as SXT, STR, NAL, AMP, TET, and AZI. Most drug-resistant strains were MDR and carried multiple resistance genes and virulence factors. *S. sonnei* and *S. flexneri* did not show particularly diverse sequence types (STs), indicating their genetic conservation and strong adaptability. Their close phylogenetic positions with strains from neighboring regions or foreign countries suggest widespread transmission. Therefore, future prevention and control of *Shigella* in this region should focus on monitoring drug resistance, targeting key populations, preventing domestic and international transmission risks, and developing vaccines against dominant serotypes.

## Figures and Tables

**Table 1:** Annual distribution of *Shigella* infections and serogroups in stool specimens from diarrheal patients in Pudong New Area of Shanghai, 2013–2024.

Year	2013	2014	2015	2016	2017	2018	2019	2020	2021	2022	2023	2024
Total stool specimens	1680	1867	2123	2025	1787	2021	1732	596	554	79	925	2281
*S. sonnei*	6	4	8	11	3	3	10	1	0	0	0	1
*S. flexneri*	2	0	2	2	1	1	0	0	0	0	0	0
Total no. isolates	8	4	10	13	4	4	10	1	0	0	0	1

**Table 2:** Antimicrobial resistance of *Shigella* isolates to various antimicrobial agents.

Antimicrobial Family	Antimicrobial Agents	Number of Resistant Isolates (%)			*p* Value
		Total (*n* = 55)	*S. sonnei* (*n* = 47)	*S. flexneri* (*n* = 8)	
β-lactams	AMP	51 (92.73)	43 (91.49)	8 (100.00)	1.000
	AMO/C	4 (7.27)	0 (0.00)	4 (50.00)	<0.05 *
	AMS	40 (72.73)	32 (68.09)	8 (100.00)	0.091
	CFZ	33 (60.00)	29 (61.70)	4 (50.00)	1.000
	CTX	31 (56.36)	29 (61.70)	2 (25.00)	0.067
	CFX	0 (0.00)	0 (0.00)	0 (0.00)	/
	CPM	9 (16.36)	8 (17.02)	1 (12.50)	1.000
	CXM	32 (58.18)	29 (61.70)	3 (37.50)	0.257
	CZA	0 (0.00)	0 (0.00)	0 (0.00)	/
	CAZ	5 (9.09)	5 (10.64)	0 (0.00)	1.000
	CEF	31 (56.36)	29 (61.70)	2 (25.00)	0.067
	IPM	0 (0.00)	0 (0.00)	0 (0.00)	/
	ETP	0 (0.00)	0 (0.00)	0 (0.00)	/
	MEM	0 (0.00)	0 (0.00)	0 (0.00)	/
Polymyxins	CT	0 (0.00)	0 (0.00)	0 (0.00)	/
	PB	0 (0.00)	0 (0.00)	0 (0.00)	/
Tetracyclines	TET	50 (90.91)	43 (91.49)	7 (87.50)	0.559
	TIG	0 (0.00)	0 (0.00)	0 (0.00)	/
Aminoglycosides	GEN	36 (65.45)	33 (70.21)	3 (37.50)	0.109
	AMK	0 (0.00)	0 (0.00)	0 (0.00)	/
	STR	53 (96.36)	45 (95.74)	8 (100.00)	1.000
Sulfonamides	SXT	55 (100.00)	47 (100.00)	8 (100.00)	/
Macrolides	AZI	21 (38.18)	20 (42.55)	1 (12.50)	0.136
Amphenicols	CHL	8 (14.55)	0 (0.00)	8 (100.00)	<0.05 *
	FFC	0 (0.00)	0 (0.00)	0 (0.00)	/
Fluoroquinolone	CIP	8 (14.55)	2 (4.26)	6 (75.00)	<0.05 *
Quinolone	NAL	51 (92.73)	43 (91.49)	8 (100.00)	1.000

* Bold value, *p* < 0.05. AMP, ampicillin; AMO/C, amoxicillin-clavulanic acid; AMS, ampicillin/sulbactam; CFZ, cefazolin; CTX, cefotaxime; CFX, cefoxitin; CPM, cefepime; CXM, cefuroxime; CZA, ceftazidime/avibactam; CAZ, ceftazidime; CEF, cefotiofur; IPM, imipenem; ETP, ertapenem; MEM, meropenem; CT, colistin; PB, polymyxin; TET, tetracycline; TIG, tigecycline; GEN, gentamicin; AMK, amikacin; STR, streptomycin; SXT, trimethoprim/sulfamethoxazole; AZI, azithromycin; CHL, chloramphenicol; FFC, florfenicol; CIP, ciprofloxacin; NAL, nalidixic acid.

**Table 3:** Presence of antibiotic resistance gene of *Shigella* strains.

Antimicrobial Family	Resistance Gene	Number of Resistant Isolates (%)			*p* Value
		Total (*n* = 55)	*S. sonnei* (*n* = 47)	*S. flexneri* (*n* = 8)	
β-lactams	*blaTEM-1B*	2 (3.64)	2 (4.26)	0 (0.00)	1.000
	*blaCTX-M-14*	2 (3.64)	2 (4.26)	0 (0.00)	1.000
	*blaOXA-1*	1 (1.82)	0 (0.00)	1 (12.50)	0.145
Tetracyclines	*tet(A)*	38 (69.09)	38 (80.85)	0 (0.00)	<0.05 *
	*tet(B)*	6 (10.91)	0 (0.00)	6 (75.00)	<0.05 *
Aminoglycosides	*aac(3)-IId*	24 (43.64)	22 (46.81)	2 (25.00)	0.443
	*ant(3″)-Ia*	44 (80.00)	44 (93.62)	0 (0.00)	<0.05 *
	*aadA5*	3 (5.45)	2 (4.26)	1 (12.50)	0.382
	*aph(3″)-Ib*	26 (47.27)	25 (53.19)	1 (12.50)	0.054
	*aph(6)-Id*	26 (47.27)	25 (53.19)	1 (12.50)	0.054
Sulphamides	*sul1*	3 (5.45)	2 (4.26)	1 (12.50)	0.382
	*sul2*	24 (43.64)	24 (51.06)	0 (0.00)	<0.05 *
Trimethoprim	*dfrA1*	54 (98.18)	47 (100.00)	7 (87.50)	0.145
	*dfrA12*	1 (1.82)	1 (2.13)	0 (0.00)	1.000
	*dfrA17*	3 (5.45)	2 (4.26)	1 (12.50)	0.382
Macrolides	*mphA*	0 (0.00)	0 (0.00)	0 (0.00)	/
Fluoroquinolone	*gyrA* (S83L)	53(96.36)	45(95.74)	8 (100.00)	1.000
	*parC* (S80I)	8(14.55)	1(2.13)	7 (87.50)	<0.05 *
	*qnrS1*	2 (3.64)	2 (4.26)	2 (25.00)	0.097
	*qnrB5*	1 (1.82)	1 (2.13)	0 (0.00)	1.000
	*qnrB19*	1 (1.82)	1 (2.13)	0 (0.00)	1.000

* Bold value, *p* < 0.05.

**Table 4:** The correlation between phenotype and genotype of *Shigella* strains.

Antimicrobial Agents	Phenotype: Resistant		Phenotype: Susceptible or Intermediate		Sensitivity (%)	Specificity (%)	Resistance Genes
	Genotype Resistant	Genotype Susceptible	Genotype Resistant	Genotype Susceptible			
AMP	3	48	0	4	5.88	100.00	*blaTEM-1B*, *blaOXA-1*
CTX	2	29	0	24	6.45	100.00	*blaCTX-M-14*
TET	43	7	1	4	86.00	80.00	*tet(A)*, *tet(B)*
STR	44	9	2	0	83.02	0.00	*ant(3″)-Ia*, *aph(3″)-Ib*, *aph(6)-Id*, *aadA5*
GEN	24	12	0	19	66.67	100.00	*aac(3)-IId*
SXT	54	1	0	0	98.18	/	*sul1*, *sul2*, *dfrA1*
AZI	0	21	0	34	0.00	100.00	*mphA*
NAL	52	0	1	2	100.00	66.67	*gyrA* (S83L)
CIP	5	3	1	46	62.5	97.87	*gyrA* (D87N, S83L), *parC* (S80I) *qnrS1*, *qnrB19*

AMP, ampicillin; CTX, cefotaxime; TET, tetracycline; STR, streptomycin; GEN, gentamicin; SXT, trimethoprim/sulfamethoxazole; AZI, azithromycin; NAL, nalidixic acid; CIP, ciprofloxacin.

**Table 5:** The distribution of virulence factor profiles in *Shigella* strains.

Virulence Factor Profiles		Number of Isolates	Rate(%)
VP1	*sigA*/*ompA*/*ipaH*	20	36.36
VP2	*sigA*/*ompA*/*ipaH*/*senB*	14	25.45
VP3	*sigA*/*ipaH*/*icsA*/*virA*/*ipaABCD*/*mxiA*/*pic*	8	14.55
VP4	*sigA*/*ompA*/*ipaH*/*senB*/*icsA*/*virA*/*ipaABCD*/*mxiA*	8	14.55
VP5	*sigA*/*ompA*/*ipaH*/*icsA*/*virA*/*ipaABCD*/*mxiA*	3	5.45
VP6	*sigA*/*ompA*/*ipaH*/*senB*/*icsA*/*virA*	1	1.82
VP7	*sigA*/*ompA*/*senB*/*icsA*/*virA*/*ipaABCD*/*mxiA*	1	1.82

VP, virulence factor profiles.

## Data Availability

Please contact author for data requests.
